# Medial Epicondyle Avulsion Fracture with Joint Entrapment in Children

**DOI:** 10.5334/jbsr.2180

**Published:** 2020-09-16

**Authors:** Anissa Lahfafa, François Dermesropian

**Affiliations:** 1 Université catholique de Louvain, BE; 2 Grand Hôpital de Charleroi, BE

**Keywords:** elbow fracture, pediatric, ossification center, medial epicondyle avulsion, CRITOE

## Abstract

**Teaching point:** The mnemonic CRITOE is a must-know that describes the sequence of elbow ossification center appearance and stands for capitellum, radial head, internal epicondyle, trochlea, olecranon and external epicondyle.

## Case Study

**Figure 1:**
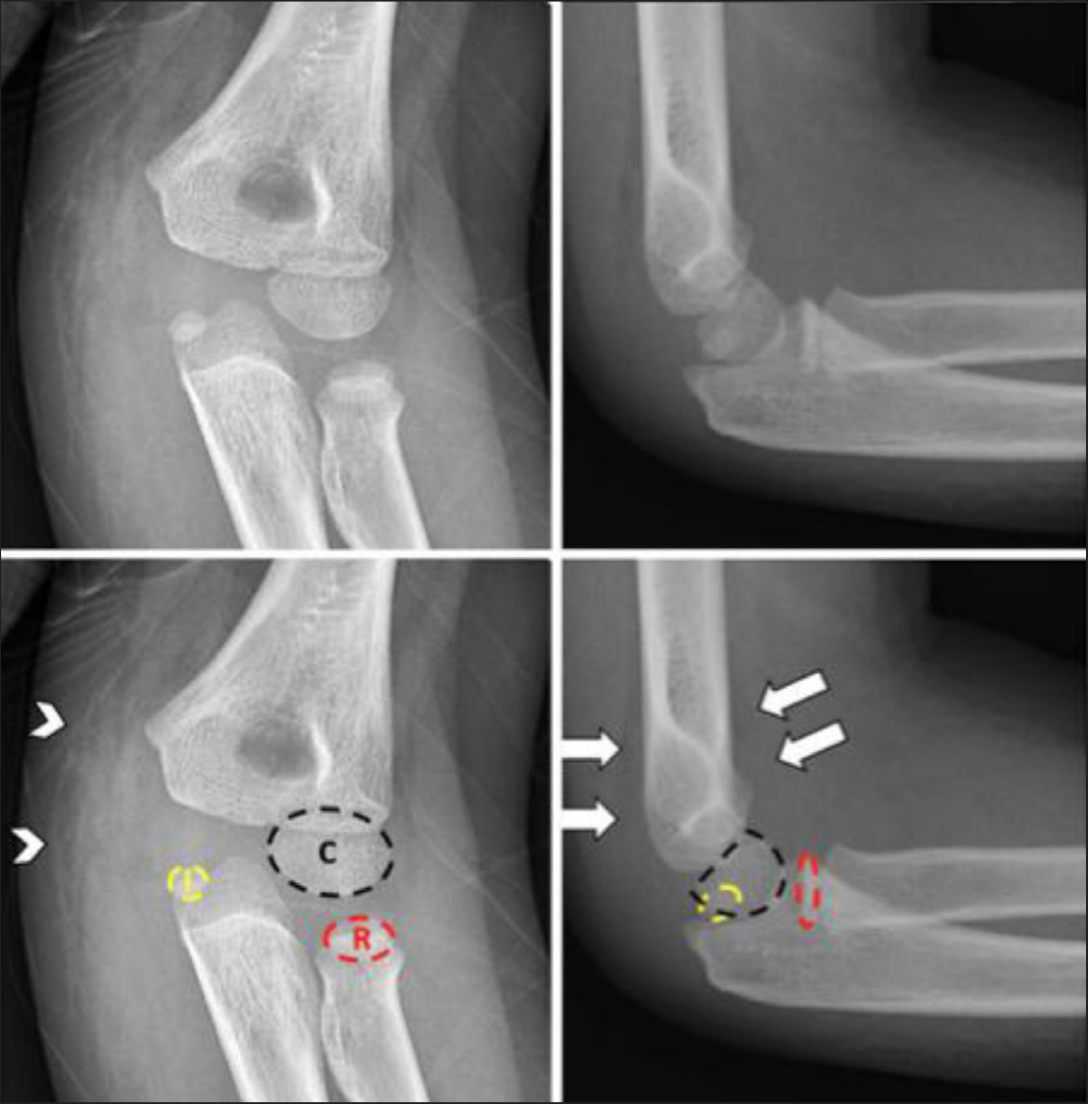


A seven-year-old boy was admitted to the emergency service for left elbow pain after a fall. On examination, his left elbow was swollen with limited movements and pain centered on the fifth ray. Diagnostic workup was completed with radiographs (Figure 1). The lateral view showed a positive fat pad sign due to joint effusion (arrows). On the frontal view, medial soft tissue swelling was noticed (arrowheads). Correct alignment between the bones was present on lateral and front views. Capitellum (C) and radius (R) ossification centers were present, contrary to the trochlea, olecranon, and lateral epicondyle. Finally, medial epicondyle (I) ossification center was displaced and entrapped in the joint between the coronoid process and the trochlea.

**Figure 2:**
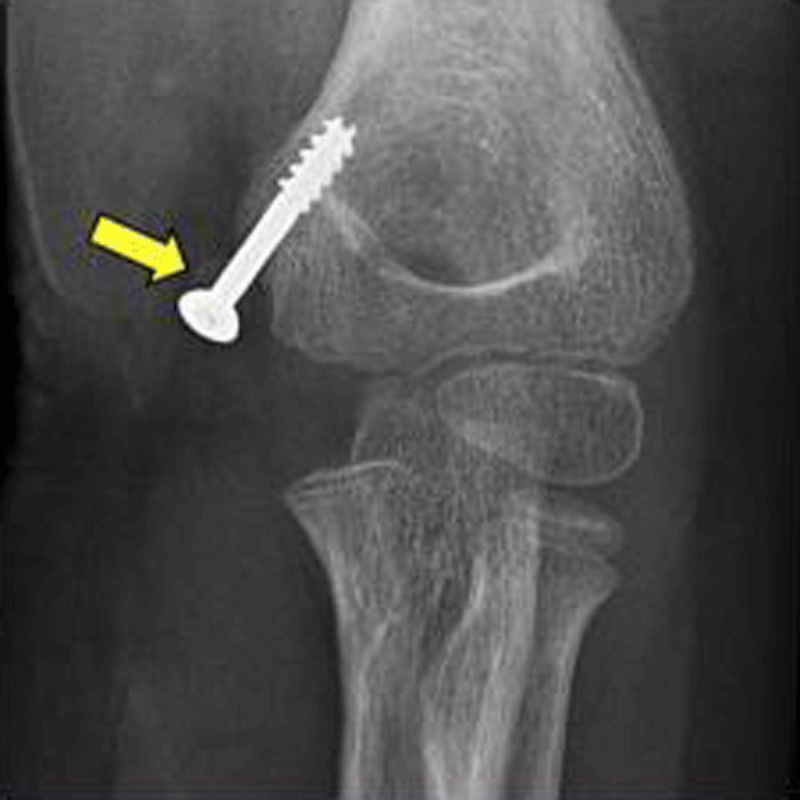


A long arm cast was placed until open reduction surgery, which allowed the release the incarcerated epicondyle fragment and its reduction osteosynthesis by a screw (Figure 2, yellow arrow). During operation, the ulnar nerve was released from the joint where it was trapped. The clinical and radiological follow-up was favorable.

## Comment

Elbow trauma in pediatric patients is very common, involving the medial epicondyle in approximately 10% of cases. It may be associated with intra-articular incarceration of the avulsed fragment, elbow dislocation, and ulnar nerve injury (due to anatomical contiguity). Radiological semiology may be challenging, especially for doctors that are not used to the ossification dynamics during childhood. There are six secondary ossification centers in the elbow, which consistently appear in an order that is key to recognition of normal anatomy [1]. CRITOE is a mnemonic for the sequence of ossification center appearance that stands for capitellum, radial head, internal (medial) epicondyle, trochlea, olecranon and external (lateral) epicondyle. Consequently, if the trochlear center is present, that of the medial epicondyle should be noticed.

Of note, ultrasound may be a valuable cost-effective tool for the assessment of partially ossified and non-ossified cartilages of the elbow in case of diagnostic uncertainties.
